# Synergistic remineralization of enamel white spot lesions using mesoporous bioactive glasses loaded with amorphous calcium phosphate

**DOI:** 10.3389/fbioe.2023.1109195

**Published:** 2023-01-19

**Authors:** Juan Ren, Jianping Rao, He Wang, Wenjing He, Jinnan Feng, Danni Wei, Bin Zhao, Xing Wang, Wei Bian

**Affiliations:** ^1^ Shanxi Medical University School and Hospital of Stomatology, Taiyuan, China; ^2^ Department of Biochemistry and Molecular Biology, School of Basic Medical Science, Shanxi Medical University, Taiyuan, China; ^3^ Shanxi Province Key Laboratory of Oral Diseases Prevention and New Materials, Taiyuan, China

**Keywords:** fixed orthodontic, remineralization, white spot lesions, amorphous calcium phosphate, mesoporous bioactive glasses

## Abstract

**Objectives:** The purpose of this study was to create a new delivery system that can synergistically remineralize enamel white spot lesions (WSLs).

**Materials and methods:** The delivery system (PAA-ACP@aMBG) was prepared by using aminated mesoporous bioactive glasses (aMBG) as the carrier loaded with polyacrylic-stabilized amorphous calcium phosphate (PAA-ACP). The materials were characterized by transmission electron microscopy (TEM), X-ray powder diffraction (XRD), inductively coupled plasma–optical emission spectrometry (ICP–OES), and so on. Forty-eight artificial WSLs enamel samples were randomized to four groups: artificial saliva (negative control, NC), casein phosphopeptide-amorphous calcium phosphate (CPP-ACP), PAA-ACP@aMBG, and MBG. The effects of demineralization and remineralization of the enamel surface were compared by means of surface microhardness (SMH) measurements, surface color change measurements, fluorescence microscopy (FM), X-ray diffraction (XRD) analysis and scanning electron microscopy (SEM).

**Results:** There was no significant difference in the surface microhardness recovery rate (SMHRR) or color recovery rate (CRR) among the CPP-ACP group, PAA-ACP@aMBG group and MBG group (P>0.05), but these values were significantly higher than those in the NC group (*p* < 0.01). FM demonstrated that the remineralization depth in the PAA-ACP@aMBG group was significantly greater than that of the remaining three groups (*p* < 0.01). SEM analysis indicated that the enamel demineralization marks in the PAA-ACP@aMBG group, CPP-ACP group, and MBG group were obscured by mineral deposition.

**Conclusions:** PAA-ACP@aMBG showed good mineralization properties, implying its great potential for clinical application.

## 1 Introduction

Orthodontic treatment can improve patient’s quality of life through aesthetic and functional improvement, but it may also damage the dental tissue (Peng et al., 2022).

White spot lesions (WSLs) caused by early enamel demineralization are one of the most frequent complications of fixed orthodontic treatment, with the incidence of at least one WSLs ranging from 36% to 46% (Chapman et al., 2010). In severe cases, orthodontic brackets may even need to be removed. The main mechanism of WSLs demineralization is the acidic erosion of plaque biofilm, which dissolves the enamel hydroxyapatite minerals (Miller et al., 2016). Therefore, remineralization therapy with hydroxyapatite reformation has become a treatment option for WSLs. This method causes calcium and phosphorus from external sources to precipitate into the micropores at the end of the enamel prism, filling the gap between the prisms caused by demineralization and restoring the mineral content in the demineralized areas (Bansal et al., 2014). The latest systematic review and meta-analysis of studies indicated that the commonly used re-mineralization agents had an unsatisfactory micro-mineralization effect, and their prevention or treatment effects on WSLs were limited (Hu et al., 2020). Therefore, the prevention and treatment of enamel demineralization is still a major challenge for dentists (Mohabatpour et al., 2022).

In recent years, amorphous calcium phosphate (ACP) has emerged as a successful precursor for the biological mineralization of dentin and bone (Qi et al., 2018; Qi et al., 2019). In light of this, a new biomineralization theory that differs from the natural enamel crystallization pathway has been confirmed (Cölfen, 2010). This theory suggests that the solution-based biomimetic mineralization system has an outstanding effect on mineralization (Wang et al., 2016). In this biomimetic mineralization system, casein phosphopeptide (CPP), polyacrylic acid (PAA), polyamidoamine (PAMAM), L-glutamic acid (L-Glu), and seven other non-collagenous biomimetic analogs play important roles in inducing ACP to remineralize type I collagen (Thula et al., 2010). Studies have demonstrated that by employing biomimetic analogs as ACP precursor stabilizers, ACP can be maintained in a moldable and nanoparticulate state, known as the polymer-induced liquid precursor (PILP) phase, and then transformed into apatite microcrystals (Yan et al., 2022). *In vitro* and animal model studies have shown that stable amorphous calcium phosphate precursors with biomimetic analogs have great potential for remineralization therapy (Ye et al., 2016; Gonçalves et al., 2021).

However, it has been reported that the existing CPP-ACP product produces a small amount of dotted and blocky high-density mineralization with uneven distribution, and a large number of demineralized pores are still visible (Fernández-Ferrer et al., 2018). It has been suggested that this is due to the rapid and uneven release rate of ACP mineralization precursors from CPP-ACP, which is dissolved and transformed into apatite microcrystals before reaching the demineralized enamel surface (Höchli et al., 2017). To solve the above problems caused by the CPP-ACP material characteristics, we introduced a novel carrier that can directly deliver CPP-ACP to the enamel surface. This material is a special mesoporous bioactive Na_2_O-CaO-SiO_2_-P_2_O_5_ glass system with good biocompatibility and surface bioactivity and a higher calcium content than the general silica-based class materials (Chen et al., 2018). It was confirmed that hydroxyapatite mineral deposits formed on the enamel and dentin surfaces after treatment with this bioactive glass (Dai et al., 2019).

At present, there are a number of reports of the use of mesoporous bioactive glass to load various metal cations (Shuai et al., 2019; Vallet-Regi and Salinas, 2021). Fang Hua et al. used mesoporous silica loaded with amorphous calcium phosphate to deliver mineralized precursors (Hua et al., 2020), Jinhua Song et al. used carboxymethyl chitosan and lysozyme to wrap amorphous calcium phosphate to construct an amorphous enamel-like layer (Song et al., 2021), and Zuohui Xiao et al. used polypeptides to induce amorphous calcium phosphate remineralization and biomimetic remineralization of dental enamel (Xiao et al., 2017). Inspired by these studies, we used an ammoniated mesoporous bioactive glass carrier loaded with polyacrylic acid-stabilized amorphous calcium phosphate PAA-ACP@aMBG. The advantage of our material is that it can achieve uniform delivery and periodic replenishment of mineralized precursors, and fully remineralized to form hydroxyapatite crystals on the enamel surface under synergistic action.

This study’s objective was to evaluate the ability of PAA-ACP@aMBG to remineralize WSLs and compare it with CPP-ACP, a commercially available remineralizer. This new material with synergistic remineralization effects is expected to be a new prospect for the treatment of early enamel caries.

## 2 Materials and methods

### 2.1 Materials

Cetyl trimethyl ammonium bromide (CTAB), calcium nitrate (Ca(NO_3_)_2_·4H_2_O), ethyl acetate, tetraethyl orthosilicate, anhydrous toluene, 3-aminopropyl triethoxysilane (APTES), polyacrylic acid (PAA), calcium chloride, disodium hydrogen phosphate, dipotassium hydrogen phosphate, glacial acetic acid, potassium hydroxide and fluoride (NaF) were all purchased from Sinopharm Chemical Reagent Co., Ltd. Artificial saliva was provided by Beijing Leigen Biotechnology Co., Ltd. All of the chemicals used in the experiments were of analytical grade. CPP-ACP dental protectors were purchased from GC Corporation Tokyo, Japan.

### 2.2 Preparation of PAA-ACP@aMBG

First, .56 g of cetyl trimethyl ammonium bromide (CTAB) was dissolved in 26 mL of deionized water and stirred vigorously at 40°C for 30 min, and then 8 mL of ethyl acetate was quickly added to the solution. Second, after adjusting the pH of the solution to 9 with ammonia, 6 mL of ethyl orthosilicate was slowly dropped into the mixture, and then 2.8 g of calcium nitrate tetrahydrate was added. Third, after stirring vigorously for 4h, the samples were centrifuged, washed three times and dried at 60°C overnight. The dried product was calcined at 700°C for 6 h to remove the structure template CTAB and obtain MBG. Finally, 100 mg MBG was dispersed in 80 mL of anhydrous toluene, and 1 mL of 3-aminopropyl triethoxysilane (APTES) was added. The mixture was condensed and refluxed for 24 h, washed three times, and then dried for 12 h at 80°C to obtain aminated mesoporous bioactive glasses (aMBG).

Add Polyacrylic acid (PAA) to a 9.0 mM CaCl_2_·2H_2_O solution to obtain a 1,000 μg/mL polyacrylic acid solution, and then 4.2 mM Na_2_HPO_4_ was added to synthesize the polyacrylate-stabilized amorphous calcium phosphate (PAA-ACP) solution. Finally, 200 mg of aMBG was added to 50 ml of PAA-ACP solution, and the mixture was stirred at room temperature for 24 h, centrifuged and dried to obtain PAA-ACP@aMBG.

### 2.3 Material characterization

#### 2.3.1 Characterization of MBG/aMBG/PAA-ACP@aMBG

X-ray powder diffraction (XRD; D8 ADVANCE A25, Germany) was used to analyze the MBG and PAA-ACP@aMBG powders. Scanning was repeated 4 times in the wide-angle diffraction range of 2θ to obtain the crystal structures of MBG and PAA-ACP@aMBG.

The chemical compositions of MBG, aMBG, and PAA-ACP@aMBG were analyzed by Fourier transform infrared spectroscopy (INVENIO, Germany). The zeta potentials of MBG, aMBG, and PAA-ACP@aMBG were measured with a zeta potential analyzer (Zeta sizer Nano, Malvern, UK). Thermogravimetric analysis (TGA) measured the load rate of aMBG from room temperature to 1,000°C under nitrogen protection at a rate of 10°C/min. The surface areas and pore sizes of MBG and aMBG were calculated using BET and BJH techniques (ASAP2020HD88 Micropore Physisorption Analyzer, United States).

#### 2.3.2 TEM evaluation of PAA-ACP@aMBG

A proper amount of PAA-ACP@aMBG was dispersed in anhydrous ethanol, followed by ultrasonic oscillation for 30 min, and a drop of solution was added to the copper net of the transmission electron microscope. After drying, solution deposition was repeated twice. TEM (JEM-2100F, JEOL, Tokyo, Japan) was used for ultra structural observation.

#### 2.3.3 ICP‒OES evaluation of PAA-ACP@aMBG

To detect whether there is a difference in the amounts of calcium and phosphorus ions released by the PAA-ACP@aMBG group at different environmental pH values, this experiment utilized three groups of solutions with different pH values: pH = 4.0 (the minimum pH value of oral bacterial acid production), pH = 5.5 (the critical value of enamel demineralization) and pH = 7.0 (the normal oral pH value); 50 mmol/L lactic acid, 50 mmol/L acetic acid and 50 mmol/L HEPES solutions were used to prepare the solutions with the above pH values. One hundred milligrams of PAA-ACP@aMBG powder were immersed in the artificial saliva solutions with different pH values, and the solutions were placed in a 37°C constant temperature shaker. Centrifugation was performed at 0.5, 1, 3, 6, 12, 24, 48, 72, 96, and 120 h. Aspirate 1 mL of supernatant and add 1 mL of fresh solution. After a 10-fold dilution, the release of calcium and phosphorus ions in solution was detected by ICP-OES.

### 2.4 Preparation of enamel samples

Forty orthodontic premolars were collected with the approval of the Ethics Committee of The School of Stomatology of Shanxi Medical University (2022SLL016). After the removal of caries, restorations, fractured teeth and teeth with surface attachments, the samples were stored in 0.1% thymol at 4°C. Each collected premolar root was removed, and the crown was divided longitudinally into buccal and lingual enamel blocks. The smooth enamel surface was obtained by grinding and polishing the enamel surface with 800, 1,000, and 1,200 particle size silicon carbide sandpaper (Shenyang, China) in flowing deionized water. Finally, the samples were ultrasonically rinsed for 15 min in deionized water to remove residues. After the samples were dried, a blank window with a size of 1 mm × 3 mm was left on the enamel surface, and the other areas were painted with acid-resistant nail polish. Vickers micro hardness tester was used to evaluate the samples surface micro hardness (SMH). Vickers micro hardness values (VHNs) > 430 or <340 were excluded. Finally, 48 samples were reserved for further experiments.

Artificial WSLs was performed by soaking the enamel samples in demineralization solution (2.2 mM Ca (NO_3_)_2_, 2.2 mM KH_2_PO_4_, 50 mM glacial acetic acid, .1 mM NaF) and adjusting their pH value to 5. The samples were demineralized at 37°C for 4 days, after that they were washed with ultrasonication in deionized water for 15 min to stop demineralization.

### 2.5 Remineralization procedure

Forty-eight artificial WSLs enamel samples were randomized to four groups (*n* = 12). Group 1 (artificial saliva, NC group): Smeared with artificial saliva twice at low speed for 1 min each time. Group 2 (CPP-ACP group): Smeared with toothpaste containing CPP-ACP (5–10 wt%) twice at low speed for 1 min each time. Group 3 (PAA-ACP@aMBG group): The dry material powder was dispersed in deionized water to obtain PAA-ACP@aMBG paste (5-10 wt%), which was coated on each sample twice at low speed for 1 min each time. Group 4 (MBG group): The dry material powder was dispersed in deionized water to obtain MBG paste (5–10 wt%), which was smeared twice at low speed for 1 min each time.

All samples underwent pH cycling for 14 days and remineralization processes performed at 08:00, 14:00, and 22:00 each day (2 min each time). The samples were soaked in demineralization solution for 1 h before remineralization treatment and then placed in artificial saliva after remineralization treatment at 37°C to simulate the oral environment. The remineralization fluid was renewed daily, and a single operator handled every operation. The samples were completely rinsed in flowing water after pH cycling, and the remaining agent was then removed using an ultrasonic rinse in flowing water for 5 min.

### 2.6 Surface microhardness measurement

Ten samples were randomly selected from each group, and their hardness was evaluated pre-demineralization, following demineralization, and following remineralization. The SMH values of all samples was determined with a microhardness tester (HV-1000A, China). The samples were loaded with a pressure of 50 g for 15 s, and the testing device automatically measured the SMH value. Five indentations were measured on each sample, with each indentation separated by at least 100 µm. The average of the five values was taken to yield one hardness value for each sample to determine the Vickers microhardness (VHN) of the sample. SMH0 denotes the enamel sample’s surface microhardness before demineralization. SMH1 denotes the enamel sample’s surface microhardness after demineralization. SMH2 denotes the enamel sample’s surface microhardness after remineralization treatment. The enamel sample’s surface microhardness recovery rate (SMHRR) was calculated using the specific formula (Lv et al., 2015): $SMHRR=\frac{\left(SMH2-SMH1\right)}{\left(SMH0-SMH1\right)}\times 100\%$ .

### 2.7 Surface color change measurement

After microhardness measurements the enamel surface color of the samples was observed using an advanced clinical portable dental spectrophotometer (VITA Easyshade® V, VITA Zahnfabrik, Bad Sackingen, Germany) and quantified by applying the CIE L*, a*, and b* values. The L* indicates the brightness from black (0) to white (100), the a* indicates red to green, and the b* indicates yellow to blue. The same lighting was used for all tests. The same operator assessed L*, a*, and b* 3 times for each sample pre- and post-demineralization and subsequent remineralization, and the mean values were recorded. Before each measurement, a white reflectance standard plate was utilized for calibration. After acquiring the L*, a*, and b* values, the color changes (∆E1 and ∆E2) before demineralization, after demineralization, and after remineralization were calculated according to the mathematical equation $\Delta E={\left[{\left(\Delta L\right)}^{2}+{\left(\Delta a\right)}^{2}+{\left(\Delta b\right)}^{2}\right]}^{1∕2}$ . Then, the formula; $CRR=\frac{\Delta E1}{\Delta E0}\times 100\%$ was used to calculate the enamel surface color recovery rate (CRR) (technology and Color, 2006).

### 2.8 FM measurements

Four samples from each group were randomly selected and sectioned vertically along the surface. The cross-sections were stained with 0.1 mM rhodamine B solution (Aldrich Chem.) for 1 h and rinsed three times under running water. Each set of samples was analyzed by fluorescence microscopy (Olympus BX53, Tokyo, Japan). The same laser power settings were used for all images. The remineralization depth (H) was then quantified by applying OLYMPUS cellSens software, and the data were averaged after three measurements by the same person.

### 2.9 XRD measurement

Four randomly selected samples from each group were scanned four times using an X-ray diffractometer (D8 ADVANCE A25, Germany) in the 2θ range to determine the crystal structure of the examined enamel surfaces.

### 2.10 SEM measurement

The remaining samples from each group were analyzed by SEM (JCM-7900F, Japan) after dehydration and gold coating.

### 2.11 Statistical analysis

The surface microhardness recovery and color recovery measurements values were tested by one-way ANOVA, and multiple comparisons were performed using the Student–Newman‒Keuls test. The remineralization depth measurements by FM were tested by Kruskal‒Wallis H-test. All data are expressed as the mean ± standard deviation (SD), and standard statistical analysis was performed using R version 3.5.3, *p* < 0.05 was considered as significant.

## 3 Results

### 3.1 Material characterization

#### 3.1.1 Characterization of MBG/aMBG/PAA-ACP@aMBG

**FIGURE 1:**
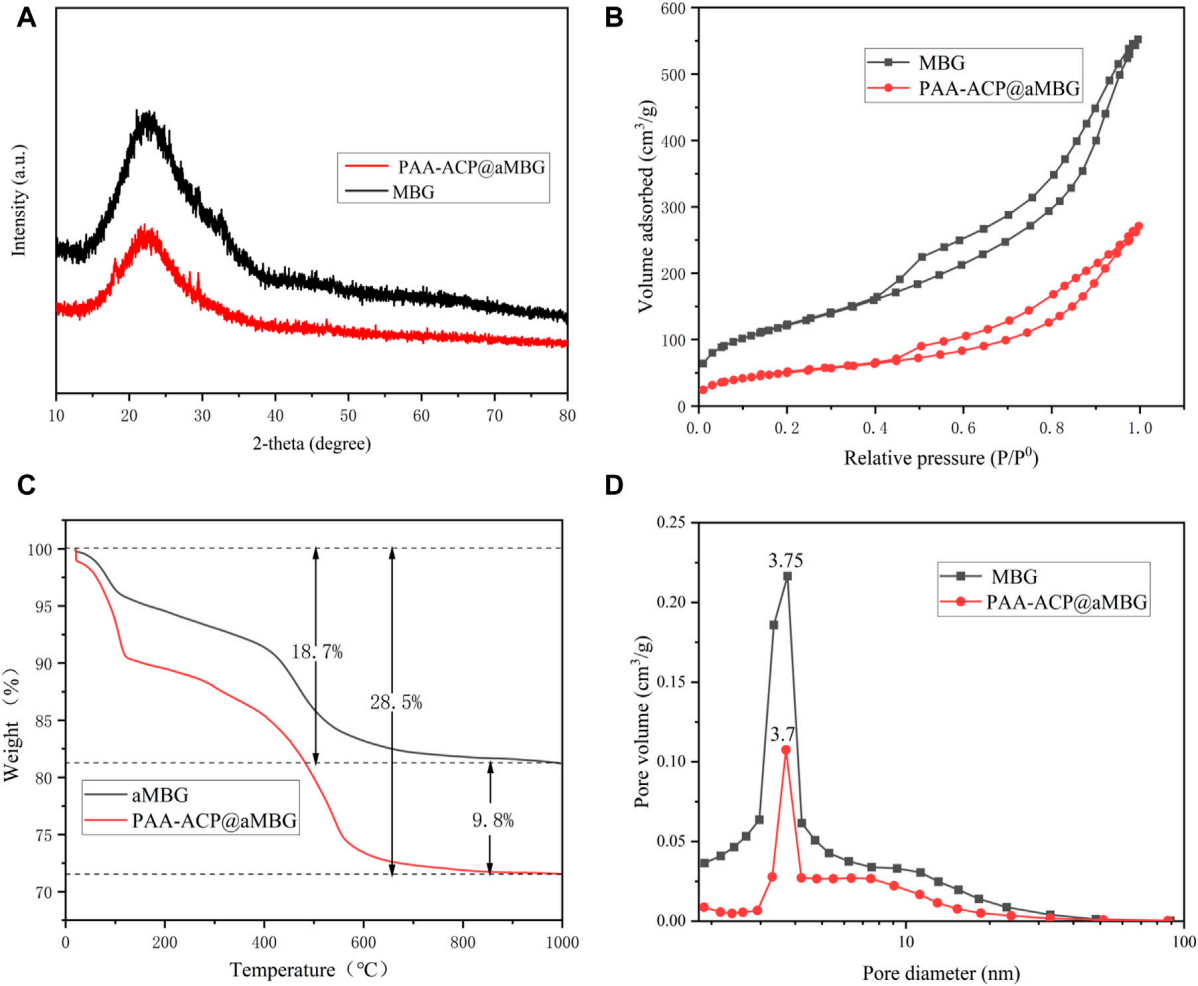
Characterization of MBG/aMBG/PAA-ACP@aMBG. **(A)** X-ray diffraction patterns of MBG and PAA-ACP@aMBG. **(B)** Nitrogen adsorption-desorption analysis of MBG and PAA-ACP@aMBG. **(C)** Thermogravimetric analysis of aMBG and PAA-ACP@aMBG. **(D)** Pore size distributions of MBG and PAA-ACP@aMBG.

Figure 1A displays the XRD patterns of MBG and PAA-ACP@aMBG. It was determined that MBG and PAA-ACP@aMBG are typical amorphous silicate glass materials, and the loading of amorphous calcium phosphate didn’t change the amorphous structure of the bioactive glass.

Figure 1C displays the thermogravimetric analysis plots of aMBG and PAA-ACP@aMBG. The total weight loss of aMBG was 18.7 wt% and that of PAA-ACP@aMBG was 28.5 wt%. The observed increase in weight loss of 9.8 wt% may be a result of PAA degradation in PAA-ACP@aMBG. This indicates that more than 9.8 wt% PAA-ACP has been absorbed onto aMBG. Nitrogen adsorption-desorption analysis (Figure 1B) showed that MBG and PAA-ACP@aMBG exhibited type IV isotherms, indicating the presence of typical mesoporous structures, with the former exhibiting H3-type hysteresis loops and the latter exhibiting H4-type hysteresis loops. The pore size, specific surface area, and pore volume decreased after MBG was loaded with amino groups and PAA-ACP (Figure 1D; Table 1).

**TABLE 1:** Specific surface areas and pore volumes of MBG and PAA-ACP@aMBG.

Samples	S_BET_ (m^2^/g)	V_P_ (cm^3^/g)
MBG	442	.85
PAA-ACP@aMBG	184	.42

S_BET_: Specific surface area; V_p_: Average pore volume.

#### 3.1.2 TEM evaluation of PAA-ACP@aMBG

**FIGURE 2:**
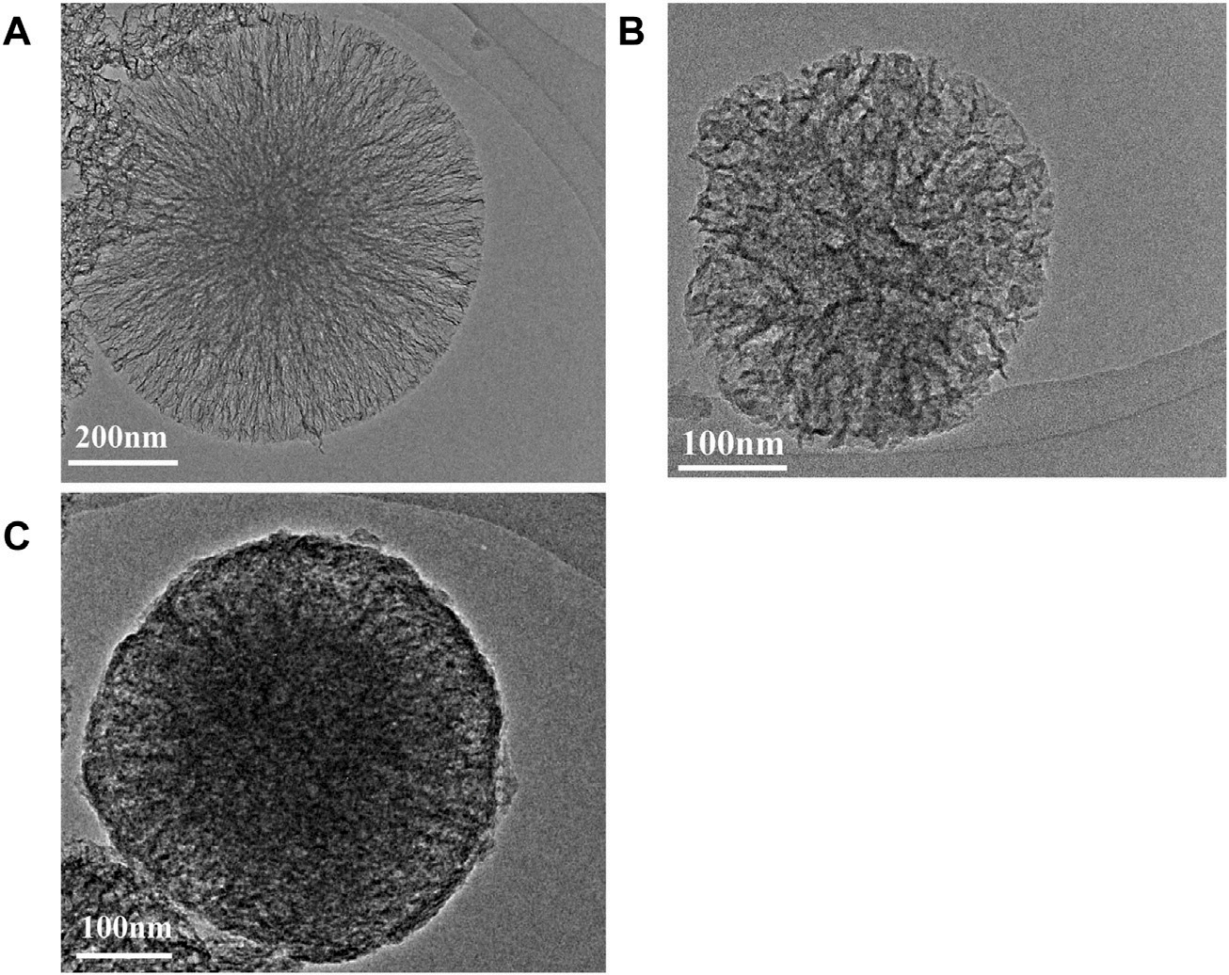
TEM images. **(A)** Bioactive glasses. **(B)** Aminated mesoporous Bioactive glasses. **(C)** PAA-ACP@aMBG.

Figure 2 displays the TEM images of MBG, aMBG, and PAA-ACP@aMBG. In Figures 2A, B, it can be seen that the mesoporous bioactive glasses and the amino acid mesoporous bioactive glasses have uniform spherical structures with an average diameter of approximately 425 nm. The mesoporous structure was relatively fluffy and ran throughout the whole mesoporous particle with an obvious hollow structure (Figure 2A). The TEM images of the mesoporous bioactive glass materials after amination showed no significant difference in their basic morphology from the mesoporous bioactive glasses, indicating that amino functionalization didn’t alter the basic structure of the materials.

After loading the PAA-ACP complex, the density of PAA-ACP@aMBG was significantly higher than that of aMBG, and the mesopores became blurred and irregular, which may be caused by blockage of the pore by PAA-ACP entry into the pore (Figures 2B, C). There were some fuzzy adsorption images on the surface of PAA-ACP@aMBG (Figure 2C), which may be caused by some PAA-ACP precursors gathering on the surface of aMBG without penetrating the interior of the mesoporous pores.

#### 3.1.3 ICP‒OES evaluation of PAA-ACP@aMBG

**FIGURE 3:**
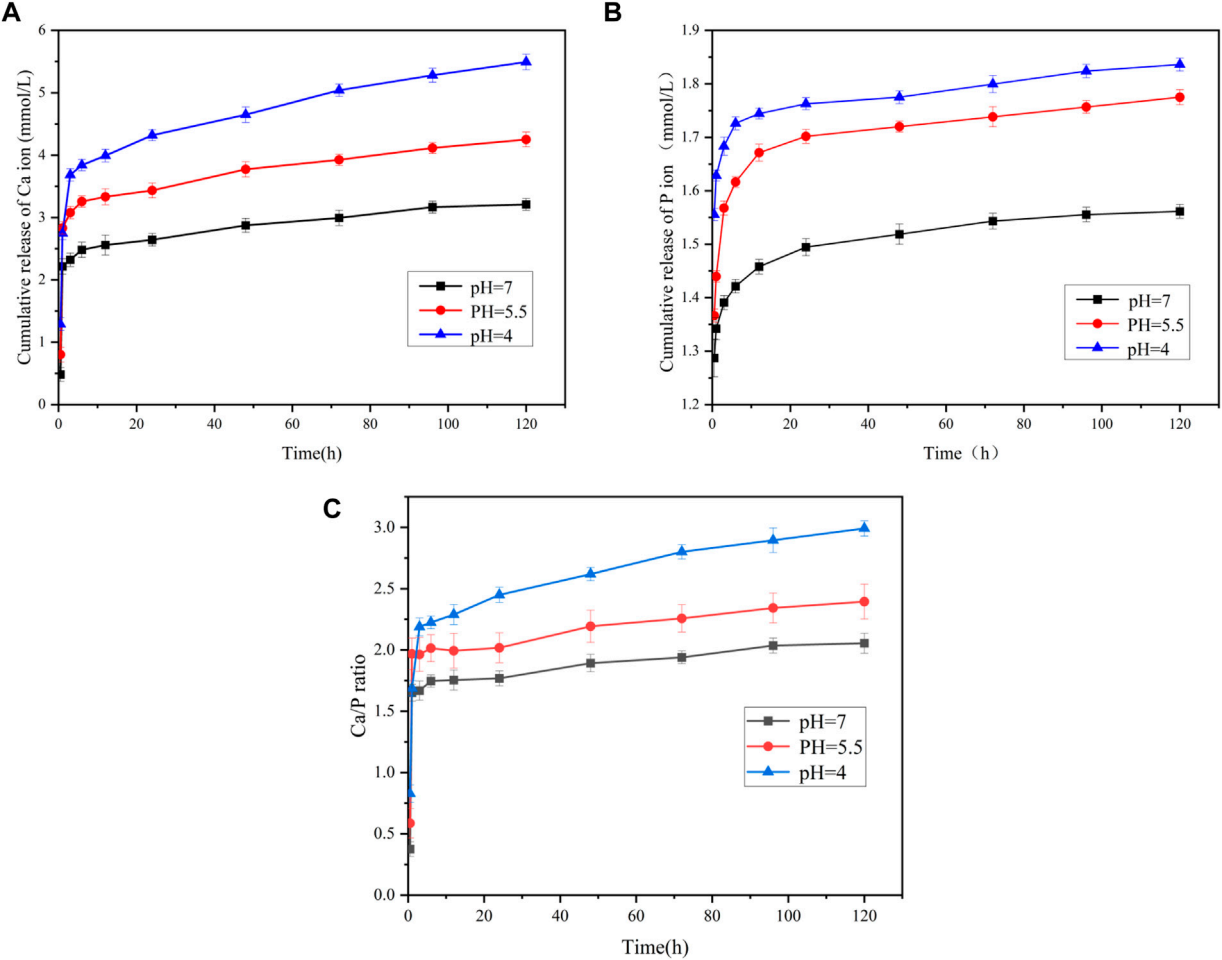
The ion release of the PAA-ACP@aMBG by ICP-OES. **(A)** Calcium ion release from PAA-ACP@aMBG. **(B)** Phosphorus ion release from PAA-ACP@aMBG under different pH conditions. **(C)** Ratio of the release of calcium and phosphorus ion from PAA-ACP@aMBG.

The release of calcium and phosphorus ions from PAA-ACP@aMBG and their ratio are shown in Figure 3. The release curves of both calcium and phosphorus ions are relatively steep at the early stage, indicating that both have a rapid release period at the early stage and then reach a smooth release period (Figures 3A, B). It is clear from the ICP‒OES figures that there is a significant difference in the release of calcium and phosphorus ions from PAA-ACP@aMBG in the same solution with different pH values. The results demonstrate that the lower the environmental pH value is, the greater the release of calcium and phosphorus ions is, and the higher the environmental Ca/P ratio is, (Figure 3C) the better the tooth hard tissues can be protected from the effects of demineralization.

### 3.2 Surface microhardness measurement

**FIGURE 4:**
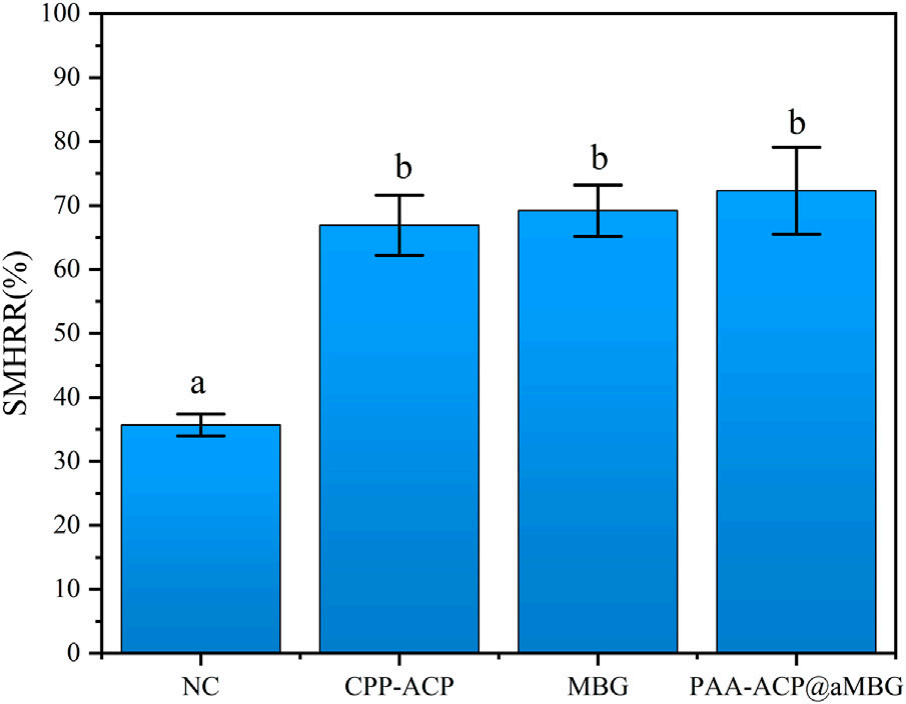
The surface microhardness recovery rates (SMHRR values) of the four groups. Identical letters indicate non-significant differences (*p* > 0.05).

Figure 4 and Table 2 display the percentage surface microhardness recovery rate (SMHRR) results. Between CPP-ACP (66.9 ± 4.7), PAA-ACP@aMBG (72.3 ± 6.8), and MBG (69.2 ± 4), there were no significant differences (*p* > .05). However, their SMHRR values were significantly higher than that of the NC group (35.7 ± 1.7; *p* < .001 for all three groups).

**TABLE 2:** SMH values (mean ± SD, VHN) and SMH recovery rates (mean ± SD, %) of the four groups at different time points (*n* = 10).

Group	Baseline	Before remineralization	After remineralization	SMH recovery ratio (%)
PAA-ACP@aMBG	385.66 ± 25.53	218.5 ± 30.25	339.42 ± 25.69	72.3 ± 6.8^b^
CPP-ACP	386.66 ± 18.64	224.83 ± 26.99	333.33 ± 13.83	66.9 ± 4.7^b^
NC	380.33 ± 13.03	227.27 ± 17.38	281.87 ± 18.55	35.7 ± 1.7^a^
MBG	379.3 ± 32.6	233.52 ± 27.90	334.32 ± 25.31	69.2 ± 4.0^b^

The same letter in SMH recovery ratio (%) indicates no significant difference between groups (*p* > 0.05).

### 3.3 Surface color change measurement

**FIGURE 5:**
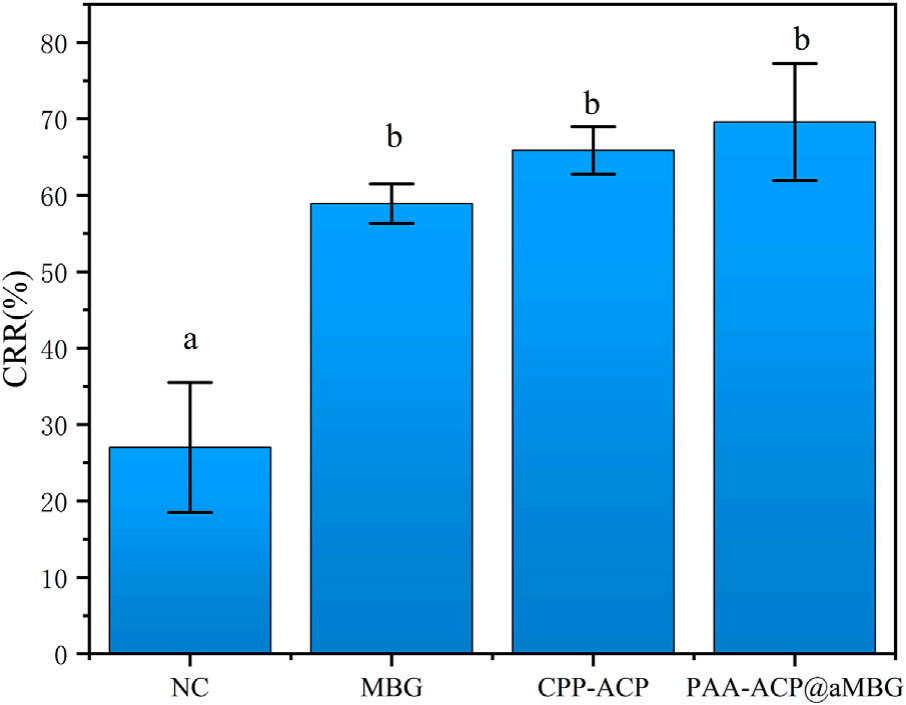
Color recovery rate (CRR) in the four groups. The same letter indicates that the difference wasn’t significant (*p* > 0.05).

Figure 5 and Table 3 display the findings of the percentage of enamel surface color recovery rate (CRR). CRR was significantly higher in the CPP-ACP (65.9 ± 3.1), PAA-ACP@aMBG (69.6 ± 7.6) and MBG groups (58.9 ± 2.6) than in the NC group (27 ± 8.5, *p* < .001). The MBG, CPP-ACP and PAA-ACP@aMBG groups didn’t differ significantly from one another (*p* > .05).

**TABLE 3:** Absolute values of color changes (mean ± SD) and color recovery rates (mean ± SD, %) for the four groups (*n* = 10).

Group	ΔE0	ΔE1	Color recovery rate (%)
PAA-ACP@aMBG	6.88 ± 1.07	4.79 ± 1.26	69.6 ± 7.6^b^
CPP-ACP	10.03 ± 2.05	6.62 ± 1.66	65.9 ± 3.1^b^
NC	8.73 ± 1.18	2.36 ± 1.02	27.0 ± 8.5^a^
MBG	9.44 ± 2.57	5.57 ± 1.77	58.9 ± 2.6^b^

The same letter in Color recovery rate (%) indicates no significant difference between groups (*p* > 0.05).

### 3.4 FM measurement

**FIGURE 6:**
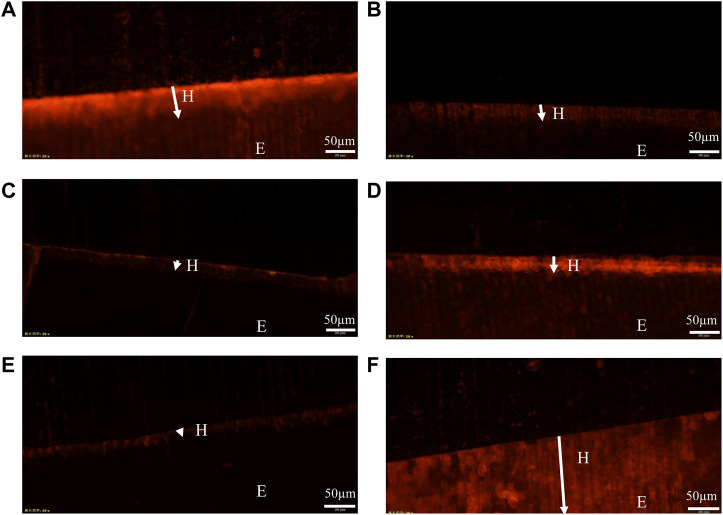
Representative FM images. **(A)**: PAA-ACP@aMBG group sample. **(B)**: CPP-ACP group sample. **(C)**: NC group sample. **(D)**: MBG group sample. **(E)**: demineralized enamel. **(F)**: normal enamel. The arrow in the figure indicates the remineralization depth (H); E represents the enamel layer.

Representative FM images are shown in Figure 6. These images reflect the fact that the fluorescent dye adsorbs to the dense remineralized enamel in the cross-section aren’t easily washed away by deionized water, whereas the fluorescent dye adsorbed on the sparse demineralized enamel in the cross-section are easily washed away by flowing water (Lena Sezici et al., 2021). So, the red remineralized fluorescence band formed in the surface layer of the enamel after remineralization represents the depth of remineralization (H). Moreover, the remineralized fluorescence band in the PAA-ACP@aMBG group (Figure 6A) was wider than that in the CPP-ACP group (Figure 6B), MBG group (Figure 6D) and deionized water group (Figure 6C), which was closer to the natural enamel group (Figure 6F). The quantitative analysis results of mineralization depth (Table 4) show that the mineralization depth of PAA-ACP@aMBG group is 62.56 ± 4.98 μm, which is significantly higher than that of other groups (*p* < 0.001).

**TABLE 4:** The depth of the enamel remineralization zone in the four groups of samples (*n* = 4).

Group	Depth (μm)
PAA-ACP@aMBG	62.56 ± 4.98^a^
CPP-ACP	46.15 ± 6.11^b^
NC	19.21 ± 1.57^c^
MBG	35.62 ± 3.76^d^

The different letter in Depth (μm) indicates significant difference between groups (*p* < 0.01).

### 3.5 XRD measurement

**FIGURE 7:**
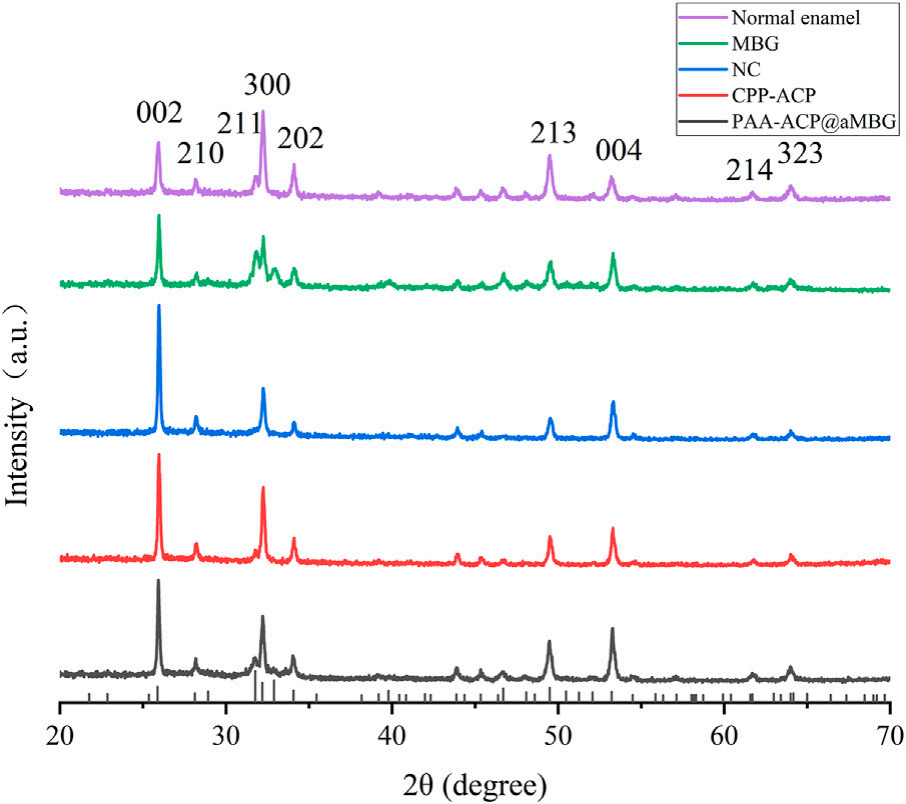
X-ray diffraction analysis of the samples. At the bottom is a standard X-ray diffraction image of hydroxyapatite (JCPDS 09-0432).

The X-ray diffraction results are shown in Figure 7. The diffractograms of the samples in the four experimental groups were identical to those of normal enamel, indicating that the minerals deposited in the four experimental groups had the same crystal structure as normal enamel. Moreover, the diffraction peaks at 25.9° (002), 28.1° (210), 31.8° (211), 32.1° (300), 34.0° (202), 49.5° (213), 53.1° (004), 61.7° (214), and 64.1° (323/304) for all four groups of samples matched the standard hydroxyapatite X-ray diffraction spectrum (JCPDS 09-0432), indicating that the sediments are mainly hydroxyapatite.

### 3.6 SEM measurement

**FIGURE 8:**
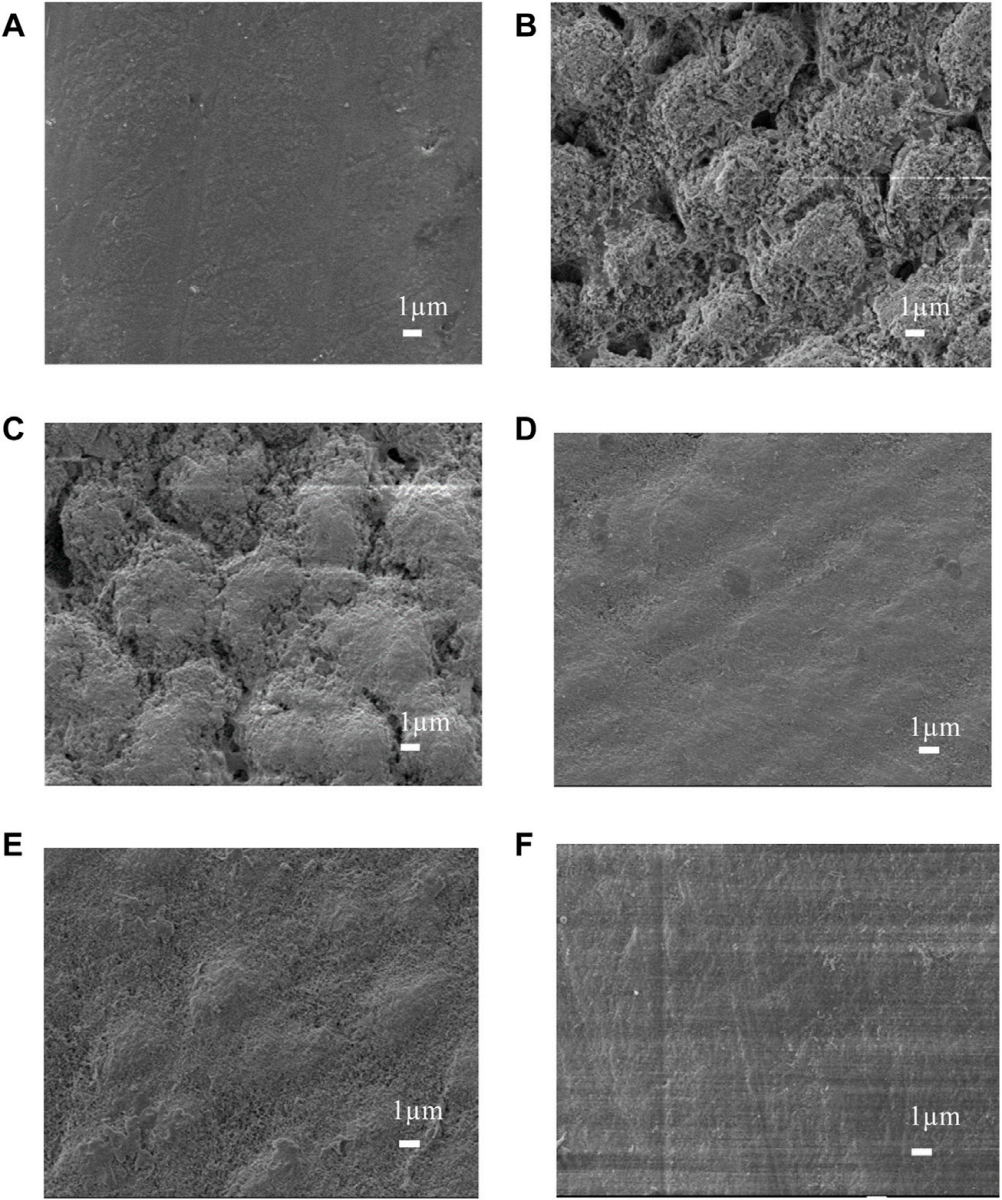
SEM images of the enamel surfaces of the four treatment groups. Images before demineralization **(A)**. Images after artificial demineralization **(B)**. Images of the nc group **(C)**. Images of the mbg **(D)**, cpp-acp **(E)** and paa-acp@ambg **(F)** groups.

Micrographs of the enamel surface are shown in Figure 8. The pictures of the normal enamel show a flat and polished surface with obvious polishing scratches (Figure 8A). Images collected following the demineralization procedure showed interprismatic gaps caused by demineralization on the enamel surface (Figure 8B). The enamel prism impressions were still visible in the NC group pictures (Figure 8C). However, in the MBG, CPP-ACP, and PAA-ACP@aMBG groups, the enamel surfaces were quite smooth, and prismatic impressions were no longer evident, showing that significant mineral deposition covered the previously visible enamel prisms and filled the interprismatic gaps (Figures 8D–F).

## 4 Discussion

Enamel WSLs are an unresolved challenge in the dental field (Sampson and Sampson, 2020). Since WSLs damage is caused by demineralization stimulated by the acidic environment of plaque biofilms, many efforts have been made to treat WSLs (Marin et al., 2022). CPP (casein phosphopeptide) is a bioactive polypeptide that can stabilize calcium and phosphorus ions in nanocomposites, thus improving the solubility and bioavailability of calcium phosphate. Therefore, CPP-ACP nanocomposites can promote remineralization by releasing these particles in response to acid attack or ion concentration changes (Philip et al., 2019). However, the distribution of CPP-ACP remineralized deposits is not uniform, and demineralized pores are still visible, which may be caused by the rapid and uneven release rate of CPP-ACP, as this material dissolves and is converted into apatite microcrystals before reaching the surface of demineralized enamel (Fernández-Ferrer et al., 2018). Comparable to CPP, PAA is a bionic analog that stabilizes ACP precursors and induces them to remineralize enamel. To achieve more homogeneous stabilization and periodic replenishment of PAA-ACP, mesoporous bioactive glasses (MBGs) would be highly desirable delivery vehicles (Feito et al., 2021). We used amino groups to functionalize MBG into aMBG, making it positively charged so that it could combine with negatively charged PAA-ACP more efficiently. In this investigation, we evaluated the synergistic remineralization ability of PAA-ACP@aMBG with deionized water as the negative control and MBG and CPP-ACP as the positive controls. Artificial demineralized enamel samples were created by immersing human premolar teeth in demineralization solutions, which is similar to procedures in previous studies (Bakry and Abbassy, 2018).

In addition, the degree of remineralization of each sample was measured by two different methods, the Vickers microhardness test and FM. The surface microhardness test has been widely used to evaluate tooth hard tissue remineralization. If the hardness increases compared to that post-demineralization, it indicates enamel remineralization and improvement in the enamel crystal structure. Moreover, FM can be used to visualize the remineralization of demineralized areas by applying rhodamine B to the demineralized areas in the cross-section. The chalky enamel caused by enamel demineralization is another major feature of WSLs, so aesthetic restoration of the demineralized areas is also necessary. The values of L*, a*, and b* utilized in this study are based on the color quantification index system established by the International Commission on Illumination (CIE) in 1976, and they are the most commonly used color system in dentistry. In this system, L* indicates the degree of light and shade, a* indicates the degree of red and green, and b * indicates the degree of yellow and blue (technology and Color, 2006). In addition, more morphological details can be obtained by observing the surface of the enamel by scanning electron microscopy. The results of the surface microhardness and surface color change tests showed that the hardness and color recovery rate in the CPP-ACP and PAA-ACP@aMBG groups were significantly higher than those in the NC group, but there was no significant difference between them (Figures 4, 5; Tables 2, 3). Our observation with the scanning electron microscope validated these findings. In the negative control group (NC), enamel imprinting and prismatic gaps were still evident, while in the CPP-ACP and PAA-ACP@aMBG pictures, these interstitial structures weren’t observed, as they were coated by precipitated minerals (Figure 8). In contrast, the FM results showed that PAA-ACP@aMBG treatment was superior to and significantly different from the CPP-ACP and MBG groups, which is strong evidence for the existence of synergistic effects between PAA-ACP and MBG.

In addition, the crystal structure of the precipitate was analyzed by X-ray diffraction. X-ray diffraction analysis is a common experimental method for identifying the crystal structures of enamel and hydroxyapatite. In this experiment, the X-ray diffraction results showed that the remineralized deposits in the four groups displayed a diffraction pattern similar to that of normal enamel, indicating that they shared a similar crystal structure (Figure 7). Moreover, comparing the diffraction peaks at 25.9°, 28.1°, 31.8°, 32.1°, 34.0°, 49.5°, 53.1°, 61.7°, and 64.1° with the standard X-ray diffraction pattern of HA (JCPDS 09-0432), these peaks were assigned to the (002), (210), (211), (300), (202), (213), (004), (214), and (323) crystallographic planes. This further indicates that the sedimentary minerals are mainly hydroxyapatite.

**FIGURE 9:**
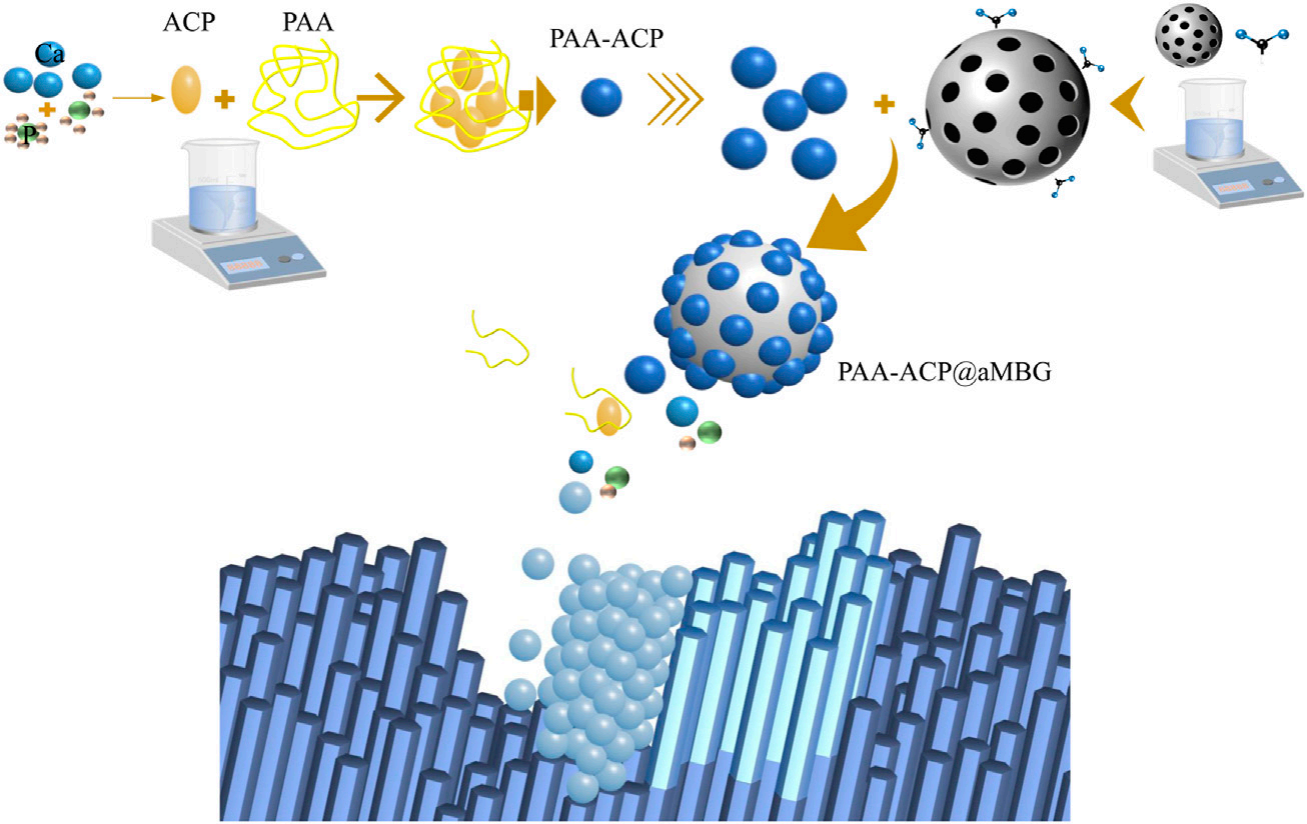
Schematic diagram.

The periodic release of PAA-ACP from MBG can be due to the competitive replacement of physically adsorbed PAA-ACP by amphoteric ions and the gradual dissolution of MBG in body fluids (Cooper et al., 2013). The possible remineralization mechanism is shown in Figure 9 described as follows. PAA-ACP particles are periodically liberated from MBG by competitive replacement of amphoteric ions and then leave under the driving force of electrostatic interactions or capillary interactions (Nudelman et al., 2010). Demineralization enamel flaws and gaps may serve as calcium and phosphorus nucleation sites, which contribute to the attachment of PAA-ACP and the subsequent release of calcium and phosphorus (Iafisco et al., 2018). Therefore, PAA-ACP can contact the surface of demineralized enamel and dissolve with MBG to liberate calcium and phosphorus. After that, these calcium and phosphorus ions can be converted into hydroxyapatite in the gap at the top of the enamel prism. Through the periodic release of calcium and phosphorus ions, complete deposition of hydroxyapatite occurs, and finally, the surface of demineralized enamel is remineralized. Although there is no significant difference between PAA-ACP@aMBG and CPP-ACP in hardness and color recovery, PAA-ACP@aMBG nanocomposites have other advantages. First, MBG has excellent mechanical properties and can be used as a nanofiller in adhesive resins with promising applications (Balbinot et al., 2020). Second, while loading PAA-ACP, aMBG can also adsorb additional nanoparticles, including antibacterial metal cations or protein repellents, thus making the delivery system multifunctional (Jiménez-Holguín et al., 2020). Moreover, after testing, it was found that PAA-ACP@aMBG could respond to pH changes in the environment and increase the release of calcium and phosphorus ions as the simulated intraoral pH decreased, predicting that PAA-ACP@aMBG would be a very suitable remineralization material for the intraoral environment.

## 5 Conclusion

In this study, PAA-ACP@aMBG nanocomposites were successfully prepared. The experimental results show that the nanocomposite can effectively remineralize enamel WSLs *in vitro* and has good color recovery performance. It has thus been shown that this material provides a new strategy for the prevention and treatment of WSLs.

## Data Availability

The original contributions presented in the study are included in the article/Supplementary material, further inquiries can be directed to the corresponding authors.
